# Using Machine Learning and Feature Importance to Identify Risk Factors for Mortality in Pediatric Heart Surgery

**DOI:** 10.3390/diagnostics14222587

**Published:** 2024-11-18

**Authors:** Lorenz A. Kapsner, Manuel Feißt, Ariawan Purbojo, Hans-Ulrich Prokosch, Thomas Ganslandt, Sven Dittrich, Jonathan M. Mang, Wolfgang Wällisch

**Affiliations:** 1 Medial Informatics, Friedrich-Alexander-Universität Erlangen-Nürnberg (FAU), 91058 Erlangen, Germany; 2 Institute of Radiology, Universitätsklinikum Erlangen, Friedrich-Alexander-Universität Erlangen-Nürnberg (FAU), 91054 Erlangen, Germany; 3 Institute of Medical Biometry, University of Heidelberg, 69117 Heidelberg, Germany; 4 Department of Paediatric Cardiac Surgery, Universitätsklinikum Erlangen, Friedrich-Alexander-Universität Erlangen-Nürnberg (FAU), 91054 Erlangen, Germany; 5 Department of Pediatric Cardiology, Universitätsklinikum Erlangen, Friedrich-Alexander-Universität Erlangen-Nürnberg (FAU), 91054 Erlangen, Germany; 6 Medical Center for Information and Communication Technology, Universitätsklinikum Erlangen, 91054 Erlangen, Germany

**Keywords:** risk factors, mortality, congenital heart defects (CHDs), machine learning (ML), random survival forest (RSF), eXtreme Gradient Boosting (XGB), feature importance

## Abstract

**Background:** The objective of this IRB-approved retrospective monocentric study was to identify risk factors for mortality after surgery for congenital heart defects (CHDs) in pediatric patients using machine learning (ML). CHD belongs to the most common congenital malformations, and remains the leading mortality cause from birth defects. **Methods:** The most recent available hospital encounter for each patient with an age <18 years hospitalized for CHD-related cardiac surgery between the years 2011 and 2020 was included in this study. The cohort consisted of 1302 eligible patients (mean age [SD]: 402.92 [±562.31] days), who were categorized into four disease groups. A random survival forest (RSF) and the ‘eXtreme Gradient Boosting’ algorithm (XGB) were applied to model mortality (incidence: 5.6% [*n* = 73 events]). All models were then applied to predict the outcome in an independent holdout test dataset (40% of the cohort). **Results:** RSF and XGB achieved average C-indices of 0.85 (±0.01) and 0.79 (±0.03), respectively. Feature importance was assessed with ‘SHapley Additive exPlanations’ (SHAP) and ‘Time-dependent explanations of machine learning survival models’ (SurvSHAP(t)), both of which revealed high importance of the maximum values of *serum creatinine* observed within 72 h post-surgery for both ML methods. **Conclusions:** ML methods, along with model explainability tools, can reveal interesting insights into mortality risk after surgery for CHD. The proposed analytical workflow can serve as a blueprint for translating the analysis into a federated setting that builds upon the infrastructure of the German Medical Informatics Initiative.

## 1. Introduction

With a prevalence of approximately 1.08% of all newborns in Germany [1], congenital heart defects (CHDs) are some of the most common congenital malformations. Many different diseases with different etiologies, including genetic [2] and nongenetic [3] causes, comprise CHD, however, therapy often requires interventional or surgical treatment [4]. Despite advances in the survival of cardiac patients, CHD is still the leading cause of mortality from birth defects [5] and imposes a substantial long-term burden [6]. According to the report of the German Registry for Cardiac Operations and Interventions in Patients with CHD, the overall in-hospital mortality for all surgical cases (Norwood type operations only registered since 2018) within a 6-year period (2013–2018) was 2.5%, rising to 6–10% in complex cases with multiple treatments [4]. International studies based on centers or registries have shown similar overall mortality rates ranging from 2.5 to 4.8% [7,8]. Globally, CHD-related mortality and morbidity remain significantly higher in low- and middle-income countries than in high-income countries [9]. Analysis of surgical outcomes and associated risk factors can therefore lead to shorter hospital stays [10] and reduce the long-term financial burden on healthcare systems [11,12]. The development of accurate risk prediction models is critical to improving outcomes.

While classical statistical methods such as the semiparametric Cox proportional hazards (CPH) regression for survival analysis have a well-founded theoretical–mathematical background and are well interpretable, clinicians and researchers strive to achieve various improvements by applying artificial intelligence (AI) methods in the healthcare domain, e.g., better detection and diagnosis of diseases [13]. However, a disadvantage of many AI algorithms is limited transparency regarding the underlying decision-making processes, which has led to the term ‘black box algorithms’ being coined for these systems [14]. This has recently led to the development of new statistical methods to ensure the comprehensibility and interpretability of the results generated by these AI methods. These efforts are referred to as *explainable artificial intelligence* (xAI) [15].

During the last decade, several machine learning (ML) algorithms have been adapted for modeling survival data. Moncanda-Torres et al. performed a survival analysis with two ML methods—a random survival forest (RSF) and the eXtreme Gradient Boosting algorithm ‘xgboost‘ (XGB) [16]—to predict breast cancer mortality risk in a large cohort [17], applying XBG’s implementation to perform Cox regression for survival data. For a better understanding of the models’ results, they applied the xAI method *SHapley Additive exPlanations* (SHAP) [18], which revealed interesting insights into how the different features contributed to the ML models’ decisions [17].

With respect to CHD, Du et al. recently applied XGB to predict the probability of in-hospital mortality after surgery in a large Chinese cohort of over 24,000 patients [19]. They were able to show that the ML method performed better in predicting the probability of in-hospital mortality after surgery compared to the RACHS-1 and STS-EACTS risk stratification scores [19]. Given the large number of different heart centers in Germany [4] and Central Europe, it would be interesting to link these data across institutions, hospitals, and federal states as well and apply these ML technologies in order to gain more insight into risk factors for CHD. The German Medical Informatics Initiative (MII) [20] established an infrastructure during the last 6 years with the goal of performing multicentric studies in a privacy-preserving manner in Germany.

As preparatory ground work, the aim of this feasibility study was to implement a data collection strategy and identify risk factors for mortality after surgery for CHD in pediatric patients at one German pediatric heart center. We applied ML methods and xAI to model post-surgery mortality risk and to allow for better interpretability of the results. Furthermore, the established analysis workflow could serve as a blueprint and benchmark for a future translation into a federated setting.

We start by describing the study sample (Section 2.1). Then, we explain the data collection (Section 2.2) and the data preprocessing and feature engineering steps (Section 2.3) and elaborate in detail on the ML experiments (Section 2.4), the application of the xAI methods (Section 2.5), and the statistical analyses (Section 2.6). In the Results section, we first provide details on the study sample using descriptive statistics (Section 3.1) and present the ‘feature-engineered’ dataset (Section 3.2) used to carry out the ML experiments (Section 3.3). The latter include computation of xAI measures (Section 3.3.1) and the application of a Cox proportional hazards regression model (Section 3.3.2) to compare the ML results with a standard statistical approach. We discuss our findings, relate them to the current literature, discuss the application of the utilized ML methods (Section 4), and elaborate on the limitations of the work (Section 4.1). Finally, we outline future efforts in the identification of risk factors for mortality after surgery for CHD in pediatric patients (Section 5).

## 2. Material and Methods

After designing the study and defining the inclusion and exclusion criteria, data associated with eligible patient encounters were extracted from the clinical data warehouse of the University Hospital Erlangen (UHE), and each case was allocated to one of four disease groups—*univentricular heart disease 1 (UVHD I)*, *univentricular heart disease 2 (UVHD II)*, *biventricular complex heart disease (BVHD cmplx.)*, and *biventricular simple heart disease (BVHD smpl.)*—using dedicated combinations of procedure and diagnosis codes (ICD), as defined in Supplemental Table S1. The survival outcome was determined using the status information, if a patient was discharged alive or died during the hospital stay, and the post-surgery follow-up time, i.e., the duration in days between surgery and discharge from hospital. Data extraction was performed using Structured Query Language (SQL), and the data were temporarily saved in a text file (comma-separated file format) before being imported into R software (version 4.4.2) [21], in which all further steps of this work (data preprocessing, ML experiments, computation of the feature importance, data analysis) were carried out, as described below.

### 2.1. Ethics Statement and Study Sample

This monocentric retrospective study was approved by the ethics committee of the Friedrich-Alexander-University Erlangen-Nürnberg, waiving the need for informed consent. The authors declare that this research was performed in compliance with the World Medical Association Declaration of Helsinki on Ethical Principles for Medical Research Involving Human Subjects. The study period was between the years 2011 and 2020. Inclusion criteria were hospitalization for cardiac surgery for CHD at the Pediatric Heart Center of the UHE within the study period and age at the time of the surgery <18 years. The procedure codes (OPS-codes, the German modification of the International Classification of Procedures in Medicine) that define eligible surgical corrections of congenital heart disease are provided in Supplemental Table S1. In brief, univentricular hearts (UVHs) were characterized by the presence of one rudimentary and one dominant ventricle (left or right), regardless of whether they had double-inlet left or right ventricles or a single atrioventricular inlet. To retain a sufficient sample size for the analysis, the group *UVHD I* comprised both patients undergoing the Norwood procedure, either as primary procedure or in a comprehensive stage II surgery, and single ventricle patients with PA-Banding or Shunt as palliative surgery stage I (sub-groups *Ia* and *Ib* in Supplemental Table S1). Likewise, the *UVHD II* group represented patients undergoing stage II and III of univentricular pathway palliation or BCPC, Glenn and TCPC, or Fontan surgery (sub-groups *IIa* and *IIb* in Supplemental Table S1).

Assignment of cases to the cardiac diagnosis groups comprising simple and complex biventricular heart diseases was performed according to Erikssen et al. [22] and further adjusted by the corresponding mortality group described by Jacobs et al. [23]. The key element of risk stratification, and thus classification between these two diagnostic groups was based on the corresponding in-hospital mortality risk estimate. For example, we classified a patient with surgical correction of AVSD as having simple biventricular heart disease (*BVHD smpl.*) due to the low in-hospital mortality rate of 2.5%, but AVSD with TOF was classified as complex biventricular disease (*BVHD cmplx.*) [23].

Premature newborns and newborns with a birth-weight <2500 g with persistent ductus arteriosus (PDA) ligation as the only cardiac procedure were excluded from the dataset (newborns with birth-weights <2500 g not matching the before-mentioned criteria were not excluded). Furthermore, to ensure a complete heart surgery history for all patients, in the present study we included only the most recent available hospital encounters (cases) for patients who were born within the study period (date of birth ≥1 January 2011). The next subsection lists the data elements that were included in this analysis.

### 2.2. Data Collection

The dataset included patient-related (sex, weight, height), encounter-related (year of the hospital admission, days until discharge after surgery), disease-related (heart disease group, deceased status [censored/deceased], associated malformations, syndrome association, presence of chromosomal alterations, presence of pulmonary hypertension), surgery-related (duration of the surgery, duration of bypassing with a heart lung machine [HLM], duration of a circulatory arrest, hypothermia, duration of using an aortic cross clamp, if the thorax was left open after surgery), and post-surgery related data elements. The latter group comprises measurements of laboratory values observed within 72 h after surgery. Further details on the definitions of disease-related features are reported in Supplemental Table S2. In order to prepare the collected data for the ML experiments and the statistical modeling, data preprocessing and feature engineering steps were required, which are described in the following subsection.

### 2.3. Data Preprocessing and Feature Engineering

For the experiments in this work, the dataset was partitioned by 60% to 40% into a training dataset and a holdout test dataset. Missing values were imputed with multivariate imputation by chained equations using the ‘mice’ R package [24], with further details given in the supplemental methods. The engineering of new features was performed after imputation of missing values.

To incorporate information on each patient’s hospitalization history, (A) the number of previous CHD-related hospital admissions with heart surgery-related procedure codes was recorded for each case in a new ordinal feature (*No. of previous hospitalizations*), and (B) the most severe heart disease group (as defined in the Methods section) that was available in the hospitalization history was recorded likewise in the feature *Heart disease history*. If previous hospitalizations for CHD surgery were present in a patient’s history, the heart disease groups were ranked by severity (in decreasing order), as follows: *UVHD I*, *BVHD cmplx.*, *UVHD II*, and *BVHD smpl*. Due to a high correlation of the weight variable and the age at surgery, body weight below <2500 g was coded in a binary manner, and the continuous weight variable was removed from the dataset. Furthermore, the height variable was removed from the analysis, as it was also strongly correlated with age at surgery. Age at surgery was kept in the dataset in favor of age at hospital admission, as they were also highly correlated with each other. Furthermore, the duration of the hospital stay was also removed from the dataset, as it is a linear combination of the days between hospital admission and surgery and the days until discharge after surgery, the latter being the time component of the survival outcome. For each laboratory value observed within a 72 h post-surgery period, the parameter that is typically associated with a more severe clinical condition was kept in the dataset, i.e., the maximum levels for serum creatinine, urea, and C-reactive protein. In contrast, for leukocytes, the minimum values were kept for further analyses as an indicator for leukopenia. For the remaining continuous features, the absolute Pearson’s correlation coefficient was <0.7. Finally, n = 19 independent variables were used to train the ML methods. The survival outcome was formed by the status variable and the post-surgery follow-up time, i.e., the duration in days between surgery and discharge from hospital (which equals the duration between surgery and death for deceased patients). Outlier cases observed with regard to the post-surgery follow-up time were censored at the 99.5% quantile of the post-surgery follow-up time, which was computed using the whole cohort. The as-such prepared data elements then served as input data for the ML experiments, which are described in the next subsection.

### 2.4. Machine Learning Experiments

Two ML algorithms that are known for their outstanding predictive abilities in modeling survival data were used throughout this analysis, namely an RSF using the implementation from the ‘ranger’ [25] R package (version 0.16.0) and XGB using the implementation from the R package ‘xgboost’ (version 1.7.8.1) [26]. Especially in the medical domain, transparency is required for clinical decision support systems so that users are able to understand the systems’ suggestions [27]. Both algorithms, RSF and XGB, belong to the family of ‘tree-based models’, which (at least to some extent) can be more interpretable as compared to other ML methods [28]. To make the results of the applied ML methods comparable with each other, predictions were obtained as risk scores. These scores can be used to rank observations by their mortality risk, even if predictions from different algorithms are on different scales. Predicting risk scores further allowed the use of Harrell’s concordance index (C-index) [29] as an evaluation metric, which is commonly used to assess prognostic models in survival analyses.

First, the hyperparameters were optimized on the training dataset with three-fold cross validation (CV). A Bayesian hyperparameter optimization was employed using the R package ‘ParBayesianOptimization’ [30]. For all experiments, Bayesian optimization was parameterized with 128 sampling runs and with the parameter Kappa of the upper confidence bound set to κ=3.5. Its default value is κ=2.576, corresponding to the ~99th percentile of the upper confidence bound with higher values allow to increase the unexplored search space. For each ML method, the Bayesian process was initialized with 50 randomly chosen parameter settings from a pre-computed parameter grid. For XGB, the parameters optimized with the Bayesian process in this study included the maximum depth of a tree (‘max_depth’), the step size shrinkage used in each update (‘learning_rate’), the subsample ratio of the training instances (‘subsample’), the subsample ratio of columns when constructing each tree (‘colsample_bytree’), and the minimum sum of instance weight that is required in a child (‘min_child_weight’). The number of rounds for boosting (‘num_round’) were optimized using ‘early stopping’, a technique that stops the learning process if no further improvement of the validation metric is observed for a pre-defined number of training iterations. Early stopping was set to 500 iterations in this study. The parameters optimized for the RSF were the number of trees (‘num.trees’), the maximal tree depth (‘max.depth’), the number of variables available for splitting in each node (‘mtry’), the minimum node size (‘min.node.size’), and the fraction of the training instances (‘sample.fraction’). For comparability, the hyperparameters of the two ML methods were optimized on identical CV folds, which were generated in a stratified manner based on the time variable, the status variable, and the disease group using the ‘splitTools’ [31] R package.

The as-such identified hyperparameter setting with the highest cross-validated C-index of each ML method was then validated in a 10 times repeated 10-fold CV on the training dataset. The folds of the repeated CV were also generated in a stratified manner and provided to all validation experiments. All of the trained 10×10 repeated CV models per ML method were then finally applied to predict the outcome in the holdout test dataset.

Similar to Moncanda-Torres et al. [17], we here also computed a CPH regression to compare the results of the ML methods with a standard statistical method for survival analysis. Therefore, the CPH models were fitted using the ‘survival’ R package [32] following the same experimental setup as outlined above. A 10×10 repeated CV was employed using the previously computed validation CV folds, and finally, all resulting CPH models were applied to predict the outcome in the holdout test dataset. Due to the rather small number of events in the dataset, only a subset of the available features were used as independent variables for the CPH regression, namely the union set of the previously identified *n* = 5 most important features of each ML method. Additionally, one single CPH model was fitted with all training data observations to compare the CPH results of the 10×10 repeated CV with the statistical standard approach for modeling survival data. In order to further explore the results of the ML methods, model explainability algorithms were employed, which are described below.

### 2.5. Model Explainability Using SHAP

The *SHapley Additive exPlanations* (SHAP) framework for interpreting model predictions was published in 2017 by Lundberg and Lee [18]. SHAP has a solid theoretical foundation based on Shapley values that were originally proposed in a game theory context by Lloyd S. Shapley [33]. These values show the magnitude of a feature’s influence on the model prediction with respect to the base-level average. When applied in a local explanation setting, SHAP values can provide insights into how a specific observation’s feature values contribute to the model’s prediction with respect to a base-level prediction. By combining the local explanations of many observations in a dataset, global insights into the functioning of a model can be gained [34]. SHAP values can be computed efficiently for tree-based models using the TreeSHAP algorithm [34]. While this algorithm is already implemented in ‘xgboost’, SHAP values for ‘ranger’ models can be computed with the ‘treeshap’ [35] R package.

When performing ML experiments, the application of resampling strategies such as CV or repeated CV is indispensable for decent model evaluation [36]. For a global model explanation in this study, SHAP values were computed for all observations in the independent holdout test dataset using each of the 10×10 repeated CV models per ML method. The results were then aggregated by averaging the SHAP values for each observation and feature across all 100 repeated CV models per ML method in the following manner:

- Let i:{i=1,2,...,n} be the i-th observation in the survival dataset.
- Let $d_{j}:\{j=1,2,...,p\}$ be the j-th feature in the dataset.
- Let $\phi \left(i_{*},d_{j}\right)$ be the SHAP value of the j-th feature of observation $i_{*}$.
- Let $M_{k}:\{k=1,2,...,l\}$ be the k-th repeated CV model.

Then, the for each feature and observation, the aggregated SHAP values $\phi_{agg}$ across all repeated CV models were computed as:

(1) $$\phi_{agg}\left(i_{*},d_{j}\right)=\frac{1}{l}\sum_{k=1}^{l}\phi \left(i_{*},d_{j}\right)$$

This approach was used in favor of arbitrarily selecting one out of the 100 models to extract the feature importance values, e.g., by selecting the model with the highest evaluation metric or—more robust—by selecting the model whose mean or median evaluation metric was closest to the overall mean or median evaluation metric of the repeated CV experiment. The aim of aggregating SHAP values across all repeated CV models was to ensure that all information generated by the repeated CV experiments were incorporated into the feature importance measures.

Furthermore, we applied the algorithm *Time-dependent explanations of machine learning survival models* (SurvSHAP (t)), a generalization of SHAP to survival models proposed by Krzyzinski et al., to compute time-dependent feature importance for survival models [37]. This algorithm overcomes the limitation that applying explanation methods, which were originally intended for standard regression and classification tasks (such as SHAP) to survival models (such as CPH), results in losing some of the importance information originating from the survival function. As this algorithm was only available for local explanations [37], we proposed an extension to aggregate SurvSHAP(t) values for each feature and time point across multiple observations in order to derive global SurvSHAP(t) values:

- Let $t\in \{t_{1},...,t_{m}\}$ be the times to the event of interest, where $t_{i1}<t_{i2}<...<t_{im}$.
- As defined by Krzyzinski et al. [37], $\phi_{t}\left(i_{*},d_{j}\right)$ is the SurvSHAP(t) value of the j-th feature of observation $i_{*}$ at time point t.

Then, global SurvSHAP(t) values $\phi_{gt}$ for a dataset with multiple observations can be computed as:

(2) $$\phi_{gt}\left(i_{*},d_{j}\right)=\frac{1}{n}\sum_{i=1}^{n}\phi_{t}\left(i_{*},d_{j}\right)$$

Likewise as above for SHAP, SurvSHAP(t) values were computed for all observations in the independent holdout test dataset using each of the 10×10 repeated CV models per ML method. Similarly, these results were also aggregated by averaging the SurvSHAP(t) values for each observation and feature across all 100 repeated CV models per ML method, resulting in aggregated global SurvSHAP(t) values $\phi_{agg_{t}}$:

(3) $$\phi_{agg_{t}}\left(i_{*},d_{j}\right)=\frac{1}{l}\sum_{k=1}^{l}\phi_{gt}\left(i_{*},d_{j}\right)$$

These aggregated global SurvSHAP(t) values were then used to visualize the time-dependent importance of the variables with regard to survival probability. In this study, SurvSHAP(t) values were computed only for the RSF, as in contrast to the R implementation of ‘xgboost’, the ‘ranger’ R package already allowed prediction of the survival function, which is a necessary prerequisite for the application of the SurvSHAP(t) algorithm.

### 2.6. Statistical Analysis

All experiments and statistical analyses were performed in the statistical programming language R, version 4.3.2 [21]. The experiments were computed on a 64-bit Windows 10 desktop PC with an Intel Core i7-6700 CPU @ 3.40GHz with 8 logical CPUs and 24 GB RAM. Summary statistics were computed in base R [21] and with the R package ‘DescrTab2’ [38]. Descriptive statistics include mean and standard deviation for continuous variables and relative and absolute frequencies for categorical variables. Stratified splitting of the survival dataset by means of the time variable, the status variable, and the disease group variable was performed with the ‘splitTools’ R package [31] (version 1.0.1) for both data partitioning and generating the CV folds. SHAP values were visualized using the ‘shapviz’ R package [39]. SurvSHAP(t) values were computed with the ‘survex’ R package [40].

## 3. Results

### 3.1. Sample Characteristics

A total of 1302 eligible patients (median age at hospital admission [IQR]: 159 [63 to 502.25] days) were included in this analysis, of which 50 patients (0.50 [0 to 17.25] days) were included in the disease group *univentricular heart disease 1 (UVHD I)*, 111 patients (1097 [219 to 1332] days) in the disease group *univentricular heart disease 2 (UVHD II)*, 291 patients (20 [0 to 232.50] days) in the disease group *biventricular complex heart disease (BVHD cmplx.)*, and 850 patients (169 [112 to 504.75] days) in the disease group *biventricular simple heart disease (BVHD smpl.)*. The median number of days of admission to the hospital prior to cardiac surgery was 1 (1 to 3) days. While the median duration of the whole hospital stay was 25 (14 to 55.50) days for patients with *UVHD I*, the patients in the *UVHD II* and *BVHD* groups were hospitalized for a shorter period, at 18 (9.50 to 32.50) and 8 (6 to 15) days, respectively. As expected, the *BVHD smpl.* group had the shortest median hospital stay, at 7 (6 to 11) days. Given the compelling necessity of surgical intervention in the neonatal and infant period, the median age at admission was lowest for the most severe heart disease categories, at 0.50 (0 to 17.25) days in the *UVHD I* group and 20 (0 to 232.50) days in the *BVDH cmplx.* cohort. The median time between admission and surgery varied considerably between patients with *UVHD I* and patients with *UVHD II*, at 6 (2.25 to 9.50) days and 1 (1 to 1) days, respectively. Patients with *BVHD smpl.* were admitted 1 (1 to 1) days on median before surgery, compared with 3 (1 to 7) days in the *BVDH cmplx.* cohort.

A total of 73 patients (5.6%) died during the post-surgery follow-up period (*UVHD I*: 62% [31/50 cases]; *UVHD II*: 7.2% [8/111 cases]; *BVHD cmplx.*: 9.6% [28/291 cases]; *BVHD smpl.*: 0.7% [6/850 cases]). The median post-surgery follow-up period (i.e., days between surgery and discharge from hospital) was 17 (11 to 48.75) days for patients with *UVHD I* and 14 (8 to 30.50) days for patients with *UVHD II*, whereas the follow-up periods for patients with *BVHD cmplx.* and *BVHD smpl.* were 10 (7 to 17) and 6 (5 to 8) days, respectively. Further patient characteristics and additional parameters, including disease-related information, are given in Table 1.

As some cases in our cohort had very long in-hospital follow-up times, with one patient being discharged 260 days after the surgery, to address potential biasing of the results, outliers with follow-up times above the 99.5% percentile, which corresponded to 130 days until discharge after surgery, were censored at that time point (see also Supplemental Figure S1). This affected a total of seven outlier cases, including *n* = 1 event.

As outlined in the methods section, only the most recent CHD-related hospitalization for each patient was included in this analysis. However, to incorporate information on each patient’s hospitalization history, the most severe heart disease group that was recorded in prior hospitalizations (which were available from the hospital information system) was added as a new feature *heart disease history* in this analysis. Supplemental Table S3 shows the number of patients of each heart defect group stratified by *heart disease history* and deceased status. It can be seen that there were 96 patients (*n* = 5 events) in the heart disease groups *BVHD cmplx.*, *BVHD smpl.*, and *UVHD II*, which were categorized as *UVHD Ia* or *UVHD Ib* groups in previous hospitalizations.

The partitioning of the as-such prepared dataset resulted in *n* = 780 patients (60% of the cohort) that were allocated to the training dataset, of which 31 patients (*n* = 18 events) belonged to the *UVHD I* group, 65 patients (*n* = 3 events) belonged to the *UVHD II* group, 172 patients (*n* = 17 events) belonged to the *BVHD cmplx.* group, and 512 patients (*n* = 4 events) belonged to the *BVHD smpl.* group (Supplemental Table S4). The median post-surgery follow-up periods were 15 days (UVHD I), 14 days (UVHD II), 10 days (BVHD cmplx.), and 6 days (BVHD smpl.). The independent holdout test dataset consisted of *n* = 522 observations (40% of the cohort), of which 19 observations (*n* = 13 events) belonged to the disease group with *UVHD I*, 46 observations (*n* = 4 events) belonged to the disease group with *UVHD II*, 119 observations (*n* = 11 events) belonged to the disease group with *BVHD cmplx.*, and 338 observations (*n* = 2 events) belonged to the disease group with *BVHD smpl.* (Supplemental Table S4). The median post-surgery follow-up periods were 33 days (*UVHD I*), 14.5 days (*UVHD II*), 10 days (*BVHD cmplx.*), and 6 days (*BVHD smpl.*).

### 3.2. Feature Engineering and Feature Selection

As a preparation step for the ML experiments, features were engineered and selected after missing values had been imputed following the approach outlined in the Methods section. A total of 21 features were finally selected for the ML experiments, comprising 19 predictor variables and the survival outcome, which was formed by the status variable and the post-surgery follow-up time. A summary of the final dataset used for the ML experiments is given in Table 2.

### 3.3. Machine Learning Experiments

**Figure 1:**
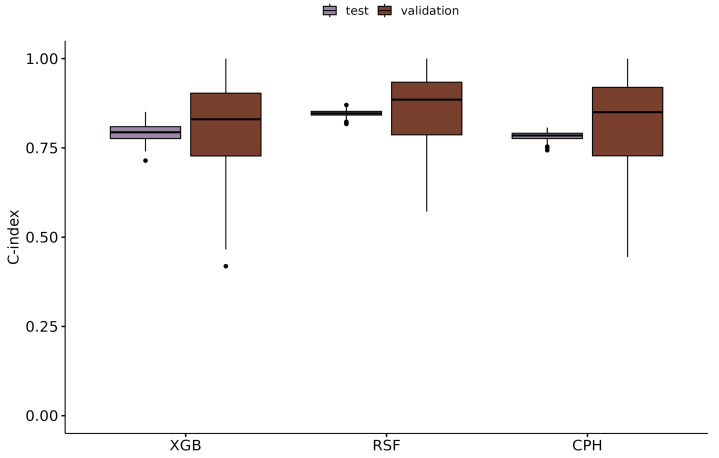
Performance: boxplots to visualize the performance of the applied models during validation and when predicting the outcome in the independent holdout test dataset. The underlying data for each boxplot are the performance of the 100 models from the repeated CV during validation and when applying these 100 models to predict the outcome in the holdout test dataset. XGB: xgboost; RSF: random survival forest; CPH: Cox proportional hazards regression.

Details on the configuration of the Bayesian hyperparameter optimization, as well as the identified hyperparameter settings for XGB and RSF, are given in Table 3. The optimal number of boosting iterations for XGB was 22. On the independent holdout test dataset, the RSF models trained with a 10×10 repeated CV achieved a C-index of 0.85 (±0.01) on average, whereas the XGB models achieved a C-index of 0.79 (±0.03) on average (Figure 1). The prediction performance of each algorithm computed with the full independent holdout test dataset, as well as stratified by disease groups, is given in Supplemental Figure S2. It can be seen that RSF and XGB performed best in *BVHD smpl.* cases, with average C-indices of 0.92 (±0.02) and 0.85 (±0.07), respectively, whereas CPH showed the best results in *UVHD II* cases, with an average C-index of 0.82 (±0.06).

#### 3.3.1. Feature Importance

**Figure 2:**
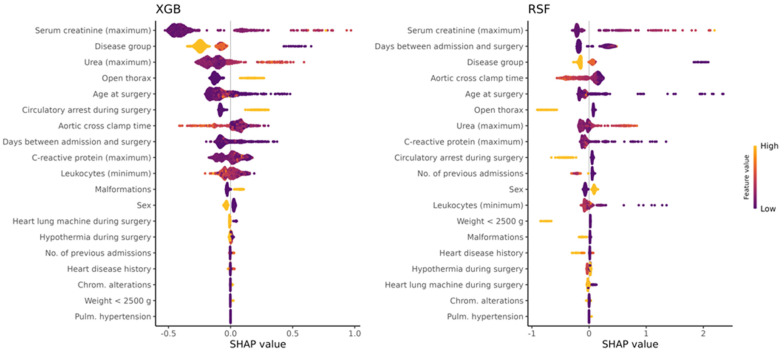
Feature importance: SHAP beeswarm plots. XGB: xgboost; RSF: random survival forest.

**Figure 3:**
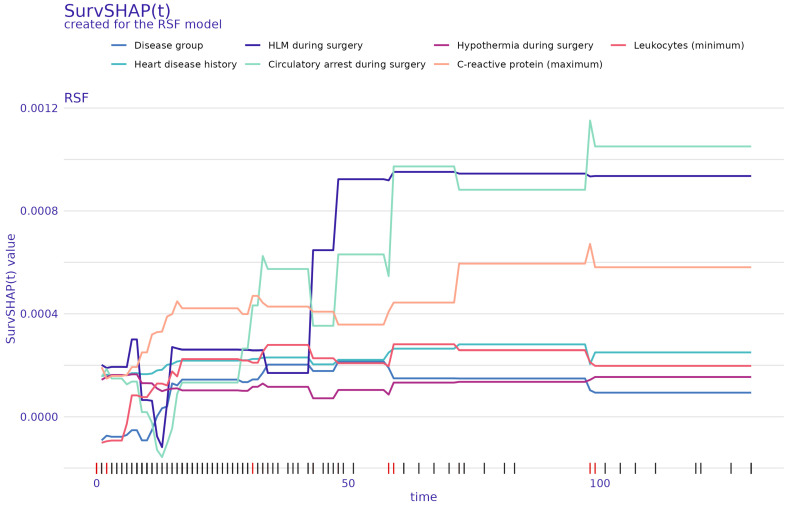
Feature importance: global SurvSHAP(t) values showing feature importance as a function of the survival time for the random survival forest (RSF). The SurvSHAP(t) values of the seven most important features identified as such were averaged by feature and evaluation time-point across all 100 repeated CV models. On the x-axis, red ticks mark event time points and black ticks mark censored time points.

For each ML method, SHAP values were computed for all observations in the holdout test dataset using all trained models from the respective 10×10 repeated CV. The global SHAP values averaged by feature and observation across all of the respective 100 CV models are visualized with beeswarm plots in Figure 2 for XGB (left) and RSF (right). The corresponding mean absolute SHAP values are given in in Supplemental Table S5. For the two ML methods XGB and RSF, SHAP identified the maximum values of *serum creatinine* observed within 72 h post-surgery as the most important feature (Figure 2). Also, the post-surgery observed maximum values of *urea* and the *age at surgery* were of high importance for both algorithms (Figure 2). The beeswarm plots further indicate that, in addition to the disease group, the *aortic cross clamp time*, the maximum post-surgery values of *C-reactive protein*, *days between admission and surgery*, and if *circulatory arrest* was applied during surgery were ranked by SHAP among the ten most important features for both ML methods to predict the outcome in the holdout test dataset (Figure 2). The binary variable if the thorax was left open after the surgery was also a notable feature ranked fourth for XGB and sixth for RSF. In comparison, for RSF, Figure 3 shows the global SurvSHAP(t) values averaged by feature, observation, and time-point across all repeated CV models. These results also take the time dependency of the feature importance into account. The union set of the five most important features of XGB and RSF according to SHAP comprises the data elements *age at surgery*, *aortic cross clamp time*, *days between admission and surgery*, *disease group*, *open thorax*, *serum creatinine (maximum)*, and *urea (maximum)* (see Table 4).

**Figure 4:**
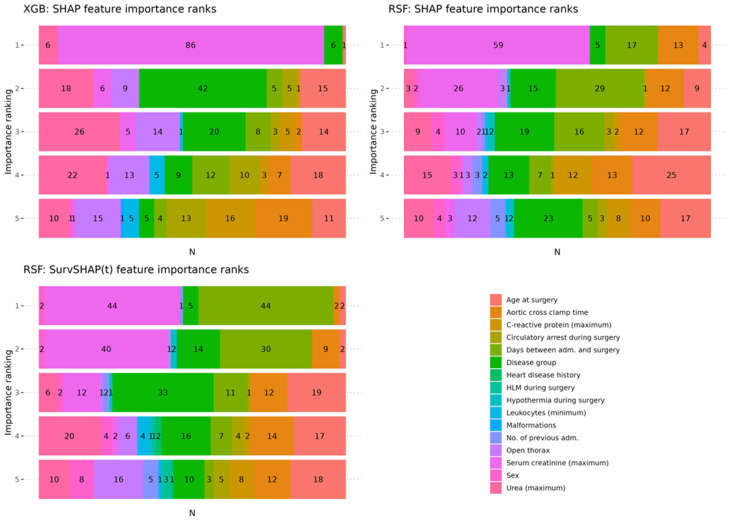
Feature importance ranks: counts of the occurrence of variables within the five most important features as defined by their mean absolute SHAP values and SurvSHAP(t) values, respectively, for each repeated CV model. XGB: xgboost; RSF: random survival forest.

To provide more insights on the feature importance variability among the repeated CV models of each ML method, Figure 4 visualizes the number of CV models in which a certain feature occurred within the five most important features according to SHAP for XGB (top left) and RSF (top right), as well as for RSF according to SurvSHAP(t) (bottom left). This visualization shows that the maximum values of *serum creatinine* observed within 72 h post-surgery was ranked according to SHAP the most important feature in 86% of all XGB models and in 59% of all RSF models. Likewise, *serum creatinine* was also ranked as the most important feature according to SurvSHAP(t) in 44% of all repeated CV models, which was on par with the *days between admission and surgery* (Figure 4). Similar to SHAP, the *disease group*, *age at surgery*, and *open thorax*, but also the post-surgery minimum values of *leukocytes* and the maximum values of *urea*, were ranked amongst the five most important features in many of the repeated CV models when computing SurvSHAP(t) values.

**Figure 5:**
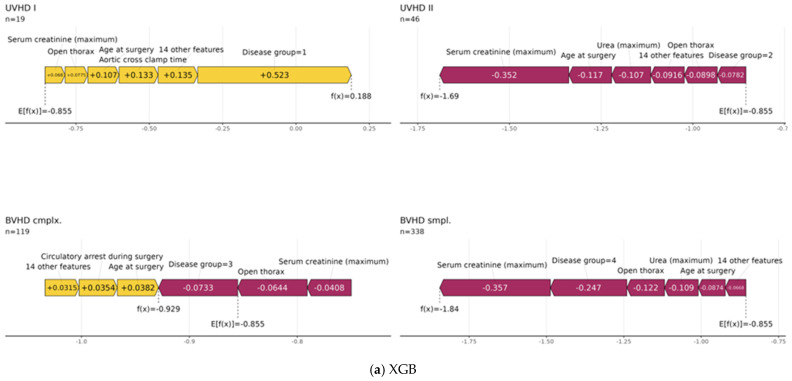

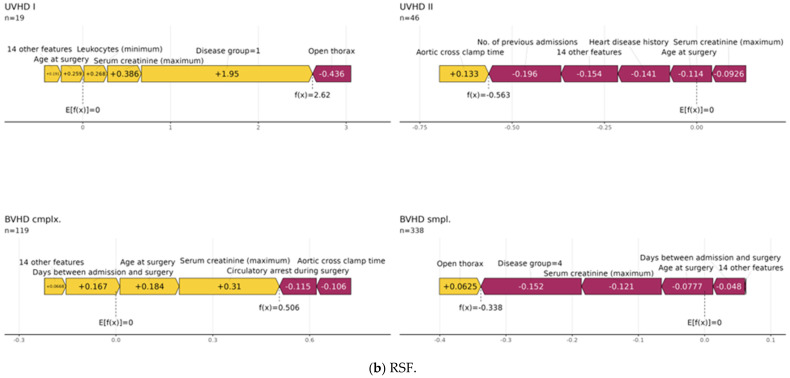
Feature importance: SHAP force plots for XGB and RSF. UVHD I: disease group univentricular heart failure (HF) 1; UVHD II: disease group univentricular HF 2; BVHD cmplx.: disease group biventricular HF complex; BVHD smpl.: disease group biventricular HF simple; XGB: xgboost; RSF: random survival forest. (**a**) XGB. (**b**) RSF.

**Figure 6:**
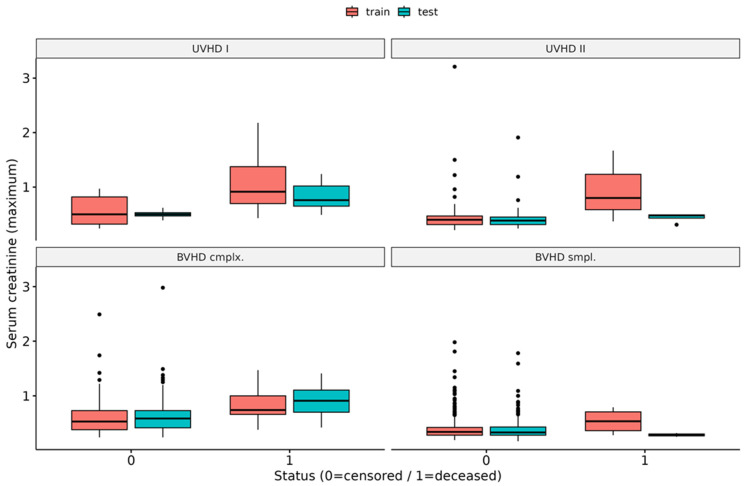
Distribution of the maximum values of serum creatinine observed within 72 h post-surgery for the heart disease groups, stratified by the training dataset and the test dataset. UVHD I: disease group univentricular heart failure (HF) 1; UVHD II: disease group univentricular HF 2; BVHD cmplx.: disease group biventricular HF complex; BVHD smpl.: disease group biventricular HF simple.

Furthermore, force plots of the aggregated global SHAP values as shown in Figure 5 for XGB and RSF are a visualization to provide more insights into the underlying data structure. Using these plots, it can be visualized how the specific observations’ feature values in the independent holdout test dataset influenced the models’ predictions stratified by the four disease groups. It is noteworthy that, according to the RSF force plots, the maximum values of *serum creatinine* noticeably seemed to increase the mortality risk on average for the disease groups *UVHD I* and *BVHD cmplx.*, whereas a negative effect of this variable was observed in the two other groups. The XGB force plots instead show an increased mortality risk of higher post-surgery maximum values of *serum creatinine* only for *UVHD I* cases, whereas in all other disease groups higher levels were associated with decreased mortality risk. Furthermore, it can be seen that the central tendency of the *serum creatinine* values is similar between the training dataset and the test dataset for the censored cases in all four disease groups. In contrast, the deceased cases in the CHD groups *UVHD II* and *BVHD smpl.* in the test dataset exhibited noticeably lower maximum values of *serum creatinine* than the corresponding training dataset (see Figure 6). Nevertheless, Figure 5 also reveals that inclusion in the disease group *UVHD I* was identified as the most important factor for increased mortality risk by both ML methods (Figure 5).

#### 3.3.2. Comparison with CPH

For comparison with a standard statistical approach, the CPH regression models were fitted in the same experimental setup as the ML methods but using only a subset of the available features. This feature subset was formed by the union set of the five most important features, as previously identified by XGB and RSF, which were used as independent variables for the CPH regression models. The CPH models achieved an average C-index of 0.78 (±0.01) in predicting the outcome in the holdout test dataset (Figure 1; for further details see supplemental results and Table S6).

## 4. Discussion

In this study, we applied a random survival forest and the ‘eXtreme Gradient Boosting’ algorithm to identify risk factors for mortality after surgery for CHD in pediatric patients at one German pediatric heart center. Both ML methods performed well in predicting the mortality risk scores in the holdout test dataset, with C-indices of 0.85 (±0.01) and 0.79 (±0.03) on average for RSF and XGB, respectively. In comparison, conventional Cox regression also performed very well, with an average C-index of 0.78 (±0.01) (Figure 1).

This is in line with other recently published studies using machine learning models for analysis of quality assessment in pediatric patients with CHD [8,19,41]. The ML methods had greater predictive power than the classical statistic methods and standard risk categories STAT and RACHS-1 in estimating in-hospital mortality, with AUCs between 0.83 and 0.88. These innovative ML algorithms appear to be ideal for complex multidimensional data and individualized risk prediction, owing to enhanced capturing of complex and nonlinear relationships [42,43].

Treatment strategies for CHD have advanced over the past decade, leading to low and consistent CHD-related overall mortality rates around 2.5% to 4.8% [7,8,44,45], depending on the patient composition of the studies. These refined surgical techniques and postoperative management have also led to paradigm shift in neonatal CHD approaches, aiming for earlier correction of complex biventricular heart defects rather than relying on palliative surgery [22]. This approach has been adopted in our center as well, which is reflected by the distribution of cardiac disease categories in this study, with about one-third of all cases being classified as complex patients, including those with *BVHD cmplx.* and *UVHD I* conditions.

We know from the literature that certain non-modifiable factors such as younger age, lower weight, and procedure type are linked to increased hospital mortality [8,46,47]. Not surprisingly, the pre-surgery risk allocation into disease groups turned out to be particularly relevant with regard to in-hospital mortality risk. This was confirmed by our xAI results, as well as by the CPH regression. In this regard it is to be noted that, at 56%, the mortality rate in the *UVHD I* group appears to be quite high in our analysis compared to the literature. However, this observed mortality rate is directly related to the cohort definition used in this analysis, where only the most recent available hospitalization for each patient was included. *UVHD* cases that were successfully discharged from hospital in the past and readmitted later, but included in a different heart defect group (due to a different surgery being performed), are consequently missing from the calculation of the *UVHD I* mortality rates, which leads to the observed higher mortality rates for this particular group in our analysis. In the study by Du et al., CHD risk categories were also identified as one of the most important features for their XGB model’s predictions [19]. While in our analysis disease groups were built by combining dedicated diagnosis and surgical procedure codes, the risk categories used in their study were based on surgical procedures and generated by clustering them into groups with homogeneous estimated mortality risks according to a dedicated statistical method [19]. Furthermore, a peculiarity in the statistical modeling of survival data is that the outcome is composed of a time variable and a status variable. The time variable indicates the total follow-up time for an observation, whereas the status indicates if an event was either observed at the end of the follow-up time frame or not, which is then referred to as *censoring*. If such data is available, it can, for example, be used for visualizing Kaplan-Meier plots and to model semiparametric CPH regression models. Hence, in our study, we have implemented the XGB algorithm using a Cox regression, which utilizes the information provided by the survival function and accounts for censoring. In contrast, Du et al. applied XGB with a binary classification [19], which is why our results are not fully comparable to theirs.

Research has highlighted the importance of an individual’s ability to handle the stress of surgery [43], which resulted in the inclusion of more patient specific pre-and postoperative variables respective complications in mortality and morbidity prediction models in cardiac surgery [45].

As part of the evolution of mortality risk models of STS–CHSD, Jacobs et al. identified pre-procedural renal dysfunction as an important contributor to increased mortality after pediatric heart surgery [23]. Cardiac surgery is a significant stressor on the kidneys, leading to pediatric acute kidney injury (AKI) in 33–43% of surgical cases [48,49], although most patients recover in a short period of time [50]. The TRIBE-AKI consortium demonstrated the importance of assessing renal function, as acute kidney injury was associated with longer mechanical ventilation, extended stays in intensive care unit (ICU), and a trend towards higher mortality [49].

Interestingly, in our analysis, the maximum creatinine level (within 72 h postoperatively) was the strongest predictor of mortality. In fact, it was ranked as the top feature in both applied ML methods, with higher feature values indicating higher risk scores (Figure 3). Global SHAP values also indicated, albeit to a lesser extent, high importance for maximum urea levels. Likewise, the influence of the maximum *creatinine* values on the mortality risk can also be seen in the SurvSHAP(t) values, with an even greater increased risk for patients with longer follow-up times (Figure 3). The importance of the post-surgery maximum *creatinine* value is further underlined by the fact that the majority of repeated CV models of both algorithms ranked it as the most important feature (Figure 4). In a subgroup analysis, the increasing effect of the maximum *serum creatinine* value on mortality risk could be shown for *UVHD I* cases and *BVHD cmplx.* cases for the RSFmodels, whereas the XGB models showed an increased risk for mortality of the higher post-surgery maximum *serum creatinine* values only in *UVHD I* cases (Figure 5). These results may be better explainable when looking at the distribution of *serum creatinine* between the different disease groups: it is noteworthy that, for the censored cases, outliers with very high values were present in all diseases groups but *UVHD I* in the training dataset and in the test dataset (Figure 6). Thus, XGB might interpret the presence of high outlier values as a protective marker depending on the heart disease group. In contrast, the figure also shows that the differences in the central tendency between censored and deceased cases in the training dataset are obvious for all disease groups. In the test dataset however, for the deceased cases, the central tendency differs from the training dataset in *UVHD II* and *BVHD smpl.* cases (Figure 6). This observation suggests that RSF may have associated this central tendency in these subgroups with a decreased mortality risk.

It should be noted that creatinine (and/or urea) levels were not a significant factor in the large database and mortality studies performed by Bertsimas et al. and Du et al. [8,19]. This, however, is different for dialysis-dependent AKI. Brown et al. demonstrated an association between this postoperative complication and increased 6-month mortality [51]. However, 30-day mortality was not affected by this complication. As the serum creatinine level was the most significant variable in risk prediction in our study, we suggest the inclusion of postoperative renal function monitoring in the form of creatinine and urea levels in future mortality prediction models.

In our study, the union set of the five most important features of the two applied ML methods XGB and RSF according to SHAP comprises the data elements *age at surgery*, *aortic cross-clamp time*, *days between admission and surgery*, *disease group*, *open thorax*, *serum creatinine (maximum)*, and *urea (maximum)*. The days between admission and surgery could reflect a worse preoperative status or admission in a decompensated cardiovascular state with the need for recovery, but this is speculative, as we did not further investigate the reason for the delayed time to surgery. The importance of preoperative status in predicting mortality was also emphasized by Bertsimas et al. in their large database study [8]. A short time (<12 days) since last admission and any common preoperative risk factor (i.e., shock, sepsis, mechanical ventilator) were associated with worse outcomes [8]. Consistent with previous studies using machine learning models for risk prediction, aortic cross-clamp or bypass time is a significant and meaningful risk factor [44,52,53]. Assessment of inflammatory state as an expression of the individual response to perioperative and postoperative factors (such as prolonged CPB) and complications demonstrated a correlation between lower postoperative leukocyte minimums and higher risk of mortality, as identified by SHAP and SurvSHAP(t) (Figure 2 and Figure 3). This laboratory value could serve as a surrogate parameter for a pronounced or abnormal inflammatory response to surgery and CPB. Systemic inflammatory response syndrome (SIRS) is common after cardiac surgery, with a rate of 20 to 32.5% [54,55] and is related to increased mortality [56]. Most commonly, however, SIRS is associated with an elevated white blood cell count [56]. We did not measure SIRS in our setting because it is difficult to accurately define and measure SIRS in the neonatal and pediatric postoperative course [57], and it ideally needs to be established prospectively. Therefore, it cannot be concluded whether this result might be related to an increased SIRS reaction.

The results of the CPH models on the independent holdout test dataset are good and almost on par with XGB. Compared to the ML methods, a big advantage when using the CPH regression is the good interpretability of its results. However, it has to be noted that, in our experimental setup, the input features provided to the CPH regression were already known to be important for the ML methods. Since these features obviously contained information relevant for the ML methods, the probability that they could also be important for the CPH regression was higher from the outset. Thus, the application of the ML methods and subsequent computation of the feature importance could be considered as kind of a (computationally very expensive) feature selection strategy for the CPH regression in our setup. Hence, the results of the CPH may not be fully comparable to those of the ML methods. Thus, using CPH might not serve well for the most fair comparison of a standard statistical method with ML methods. Therefore, for example, elastic net regression (ENR) would be more suitable, as it also includes variable selection. However, as the interpretation of CPH is more comprehensive and straightforward compared to penalized regression methods such as ENR, here we only used CPH as a base level for comparison with RSF and XGB.

### 4.1. Limitations

This study had several limitations. The major limitation is the rather low sample size, and especially the relatively low number of events, which hampered statistical modeling, especially when using ML algorithms that usually require much more data than standard statistical methods. This low number of events may have influenced the application of resampling strategies such as the CV techniques utilized here, potentially producing fold configurations without any events. The not-optimal splitting becomes obvious in the differing data distributions between the training dataset and the holdout test dataset, for example, with regard to the follow-up time periods or to the distribution of the post-surgery *creatinine* values among the different disease groups. On the one hand, we addressed this issue by partitioning the data by 60% to 40% into a training dataset and a holdout test dataset in order to ensure that enough events were available for each disease group in both data partitions. Furthermore, generation of the CV folds was stratified by disease group, time- variable, and status variable to avoid the accumulation of edge cases in a few folds. On the other hand, validation of the hyperparameters was implemented with a 10× repeated 10-fold CV to take the variability of the differently configured folds into account. Indeed, the boxplots in Figure 1 show high variability of the respective model performance among the 100 folds during the hyperparameter validation of each algorithm.

Despite all the adjustments that were made regarding the probably insufficient sample size in this study, it would be beneficial to overcome this limitation by analyzing data from multiple German pediatric heart centers jointly. One goal of this feasibility study was to establish an analytical workflow that can be used as a blueprint for designing a multicentric study in the future. During the last 6 years, the German Medical Informatics Initiative (MII) [20] established an infrastructure in German university hospitals to conduct such multicentric studies in a privacy-preserving manner. Based on this infrastructure, the Bavarian Cancer Research Center, for example, is currently establishing approaches for federated machine learning (fML).

However, we have identified several challenges that still need to be addressed in order to conduct our analysis in a federated multicentric setting. The statistical learning methods applied in this study, namely RSF, XGB, and CPH, cannot directly be applied in a federated setting. The adaption of these algorithms for fML is part of ongoing research efforts, both for RSF [58,59,60] and for XGB [61,62,63]. Similarly, current research aims at translating the statistical modeling of survival data into a federated setting [64,65,66], including federated survival forests [67], as well as an extension of CPH, so called ‘discrete-time Cox models’ [64]. To also make the outputs of the fML models better understandable and interpretable, xAI methods such as SHAP need to be translated to the federated learning setting as well, which is also currently being investigated by many research groups [68,69,70,71,72,73]. Another important aspect is the handling of missing values in the federated setting. Here, we used a multiple imputation approach, which could, for example, be implemented at each participating site separately.

A limitation of the cardiology diagnosis groups is their reliance on ICD and OPS codes, which can be misleading if not accurately coded. To test the accuracy of the algorithm, the dataset was compared to two other registries, the German national registry and the ECHSA database, which regularly receive data on surgical and interventional procedures from our center. The results showed similar numbers of cases and a comparable proportion of complex operations, confirming the accuracy of the algorithm. The wide variety of diseases, diagnoses, procedures, and combinations in the field of pediatric cardiology and congenital heart surgery makes categorization challenging. Categorization frequently fails to consider the interaction between surgical and patient-specific factors. Moreover, even within the same category, there is considerable variation in severity, which makes accurate prognosis and risk stratification challenging.

## 5. Conclusions

In this work, we applied advanced ML methods to identify risk factors for mortality after surgery for CHD in pediatric patients at one German Pediatric Heart Center. We demonstrated that ML methods can be applied in combination with dedicated model explainability tools to reveal interesting insights into mortality risk after surgery for CHD, such as the seemingly high importance of the *maximum values of serum creatinine* observed within 72 h post-surgery. With respect to clinical relevance, future efforts are needed to validate the findings and to investigate potential countermeasures. Furthermore, building upon this preparatory groundwork, future work needs to investigate if the analysis workflow established here can be translated into a federated setting in order to analyze a larger cohort.

## Figures and Tables

**Table 1:** Patient characteristics. State of the dataset before feature engineering. UVHD I: disease group univentricular heart defect (HD) 1; UVHD II: disease group univentricular HD 2; BVHD cmplx.: disease group biventricular HD complex; BVHD smpl.: disease group biventricular HD simple.

Category	Variable	UVHD I	UVHD II	BVHD Cmplx.	BVHD Smpl.	Total Cohort
Demographics	*N* (%)	50 (3.84)	111 (8.53)	291 (22.35)	850 (65.28)	1302 (100.00)
	Median age at adm. (IQR) [days]	0.50 (0 to 17.25)	1097 (219 to 1332)	20 (0 to 232.50)	169 (112 to 504.75)	159 (63 to 502.25)
	Median height (IQR) [cm]	51.50 (49.25 to 53.75)	92 (67.50 to 100)	54 (50 to 67.50)	65 (59 to 78)	64 (55 to 78)
	Missing height (%)	0 (0.00)	0 (0.00)	0 (0.00)	1 (0.08)	1 (0.08)
	Median weight (IQR) [kg]	3.30 (2.96 to 3.88)	13 (6.93 to 15)	3.96 (3.29 to 6.93)	69 (4.69 to 9)	5.81 (3.96 to 9.10)
	Missing weight (%)	0 (0.00)	5 (0.38)	3 (0.23)	21 (1.61)	29 (2.23)
	Sex: male (%)	30 (60.00)	73 (65.77)	187 (64.26)	441 (51.88)	731 (56.14)
	Sex: female (%)	20 (40.00)	38 (34.23)	104 (35.74)	409 (48.12)	571 (43.86)
Encounter-related	Median days duration of stay (IQR)	25 (14 to 55.50)	18 (9.50 to 32.50)	15 (9 to 23.50)	7 (6 to 11)	9 (6 to 17)
	Median days between adm. and surgery (IQR)	6 (2.25 to 9.50)	1 (1 to 1)	3 (1 to 7)	1 (1 to 1)	1 (1 to 3)
	Median days disch. after surgery (IQR)	17 (11 to 48.75)	14 (8 to 30.50)	10 (7 to 17)	6 (5 to 8)	7 (5 to 12)
Disease-related	Median no. of previous adm. (IQR)	0 (0 to 0)	2 (1 to 2)	0 (0 to 1)	0 (0 to 0)	0 (0 to 0)
	Deceased status (%)	31 (62.00)	8 (7.21)	28 (9.62)	6 (0.71)	73 (5.61)
	Malformations (%)	16 (32.00)	22 (19.82)	39 (13.40)	88 (10.35)	165 (12.67)
	Chrom. alterations (%)	3 (6.00)	2 (1.80)	26 (8.93)	180 (21.18)	211 (16)
	Pulm. hypertension (%)	0 (0.00)	2 (1.80)	4 (1.37)	9 (1.06)	15 (1)

**Table 2:** Machine learning dataset. State of the dataset after feature engineering. ^1^: Chi-squared test. ^2^: Wilcoxon rank sum test. UVHD 1: disease group univentricular heart defect (HD) 1; UVHD 2: disease group univentricular HD 2; BVHD cmplx.: disease group biventricular HD complex; BVHD smpl.: disease group biventricular HD simple.

Category	Variables		Censored	Deceased	Total	*p*
Demographics	Sex	m	686 (56%)	45 (62%)	731 (56%)	0.263 ^1^
		w	544 (44%)	27 (38%)	571 (44%)	
	Weight <2500 g	No	1201 (98%)	62 (86%)	1263 (97%)	<0.001 ^1^
		Yes	29 (2%)	10 (14%)	39 (3%)	
Disease-related	Chrom. alterations	No	1027 (83%)	64 (89%)	1091 (84%)	0.227 ^1^
		Yes	203 (17%)	8 (11%)	211 (16%)	
	Disease group	BVHD cmplx.	263 (21%)	28 (39%)	291 (22%)	
		BVHD smpl.	844 (69%)	6 (8%)	850 (65%)	
		UVHD I	19 (2%)	31 (43%)	50 (4%)	<0.001 ^1^
		UVHD II	104 (8%)	7 (10%)	111 (9%)	
	Heart disease history	No previous hospitalization	956 (78%)	56 (78%)	1012 (78%)	0.360 ^1^
		BVHD smpl.	52 (4%)	0 (0%)	52 (4%)	
		UVHD II/UVHD III	5 (0%)	0 (0%)	5 (0%)	
		BVHD cmplx.	123 (10%)	9 (12%)	132 (10%)	
		UVHD Ib	35 (3%)	4 (6%)	39 (3%)	
		UVHD Ia	59 (5%)	3 (4%)	62 (5%)	
	Malformations	No	1092 (89%)	45 (62%)	1137 (87%)	<0.001 ^1^
		Yes	138 (11%)	27 (38%)	165 (13%)	
	Pulm. hypertension	No	1215 (99%)	72 (100%)	1287 (99%)	0.346 ^1^
		Yes	15 (1%)	0 (0%)	15 (1%)	
Encounter-related	Days between admission and surgery	mean ± sd	3.5 ± 9.8	6.9 ± 7.5	3.7 ± 9.7	<0.001 ^2^
		min−max	0–182	0–34	0–182	
	Days until discharge after surgery	mean ± sd	12 ± 16	27 ± 28	13 ± 18	<0.001 ^2^
		min − max	1–130	0–122	0–130	
	No. of previous admissions	mean ± sd	0.32 ± 0.66	0.32 ± 0.69	0.32 ± 0.66	0.967 ^2^
		min − max	0–4	0–3	0–4	
Laboratory analytes	C-reactive protein (maximum)	mean ± sd	58 ± 44	68 ± 56	58 ± 45	0.326 ^2^
		min − max	1–356	0.1–259	0.1–356	
	Leukocytes (minimum)	mean ± sd	9.7 ± 3.5	6.9 ± 3.6	9.6 ± 3.6	<0.001 ^2^
		min − max	1.5–26	1.6–16	1.5–26	
	Serum creatinine (maximum)	mean ± sd	0.44 ± 0.26	0.85 ± 0.39	0.47 ± 0.28	<0.001 ^2^
		min − max	0.17–3.2	0.25–2.2	0.17–3.2	
	Urea (maximum)	mean ± sd	32 ± 17	48 ± 21	33 ± 17	<0.001 ^2^
		min − max	5–152	12–107	5–152	
Surgery-related	Age at surgery	mean ± sd	422 ± 568	152 ± 344	407 ± 561	<0.001 ^2^
		min − max	0–3466	0–1597	0–3466	
	Aortic cross clamp time	mean ± sd	72 ± 57	68 ± 61	72 ± 57	0.437 ^2^
		min − max	0–280	0–284	0–284	
	Circulatory arrest during surgery	No	1091 (89%)	44 (61%)	1135 (87%)	<0.001 ^1^
		Yes	139 (11%)	28 (39%)	167 (13%)	
	Heart lung machine during surgery	0 min.	165 (13%)	10 (14%)	175 (13%)	0.282 ^1^
		≥1 min–<90 min	207 (17%)	7 (10%)	214 (16%)	
		≥90 min	858 (70%)	55 (76%)	913 (70%)	
	Hypothermia during surgery	>32 °C	308 (25%)	19 (26%)	327 (25%)	<0.001 ^1^
		≥28 °C–≤32 °C	455 (37%)	12 (17%)	467 (36%)	
		<28 °C	467 (38%)	41 (57%)	508 (39%)	
	Open thorax	No	1162 (94%)	31 (43%)	1193 (92%)	<0.001 ^1^
		Yes	68 (6%)	41 (57%)	109 (8%)	

**Table 3:** Optimized hyperparameters. XGB: xgboost; RSF: random survival forest.

Algorithm	Hyperparameter	Minimum	Maximum	Step Size	Bounds	Optimized Value
XGB	colsample_bytree	0.5	0.8	0.3	[0.3, 1]	0.8
	learning_rate	0.01	0.11	0.05	[0.001, 0.2]	0.11
	max_depth	1	9	4	[1, 40]	5
	min_child_weight	1	9	4	[0, 10]	1
	subsample	0.5	0.8	0.3	[0.3, 1]	0.5
RSF	max.depth	1	9	4	[1, 40]	40
	min.node.size	1	9	4	[1, 20]	20
	mtry	2	6	2	[2, 9]	2
	num.trees	500	1000	500	[100, 1000]	100
	sample.fraction	0.5	0.8	0.3	[0.3, 1]	0.63

**Table 4:** Top features. The table shows the *n* = 5 most important features (by means of the mean absolute SHAP values) for xgboost (XGB) and ranger (RSF), respectively.

Top Features	XGB	RSF
Age at surgery	0.12	0.18
Aortic cross clamp time	n/a	0.19
Days between admission and surgery	n/a	0.24
Disease group	0.20	0.19
Open thorax	0.13	n/a
Serum creatinine (maximum)	0.38	0.31
Urea (maximum)	0.15	n/a

## Data Availability

The datasets generated and/or analyzed during the current study are not publicly available, due to internal data transfer policies, but are available from the corresponding author on reasonable request. The code used to perform the experiments is available at GitHub: https://github.com/kapsner/CHD-risk-factors (as of 14 November 2024). As no suitable R package existed for performing the desired analyses with the algorithms implemented in ‘ranger’ [25], ‘xgboost’ [26], and ‘survival’ [32], we developed a software framework consisting of the R packages ‘mlexperiments’ to perform the hyperparameter optimization, as well as the (repeated) CV, and ‘mlsurvlrnrs’ to provide some learner algorithms for survival data. The R package ‘mlexperiments’ is publicly available under open source license at The Comprehensive R Archive Network (CRAN): https://CRAN.R-project.org/package=mlexperiments (as of 14 November 2024). The R package ‘mlsurvlrnrs’ is publicly available under open source license at CRAN: https://CRAN.R-project.org/package=mlsurvlrnrs (as of 14 November 2024). Throughout this work, the computation of SHAP values for random survival models with the ‘ranger’ R package has been contributed as a new feature to the R package ‘treeshap’, which is publicly available at CRAN: https://CRAN.R-project.org/package=treeshap (as of 14 November 2024). Furthermore, the computation of SurvSHAP(t) values using the ‘treeshap’ R package as well as the computation of global SurvSHAP(t) values has been contributed as a new feature in the R package ‘survex’, which is publicly available at CRAN: https://CRAN.R-project.org/package=survex (as of 14 November 2024).

## Supplementary Materials

The following supporting information can be downloaded at: https://www.mdpi.com/article/10.3390/diagnostics14222587/s1, Table S1. Inclusion criteria. If groups are defined by both OPS and ICD codes, the respective codes are concatenated with a logical ‘AND’ between code systems (ICD/OPS) and ‘OR’ within one code system. OPS: German procedure coding system; ICD: internationial classification of diseases; UVHD I: disease group univentricular heart defect (HD) 1; UVHD II: disease group univentricular HD 2; BVHD cmplx.: disease group biventricular HD complex; BVHD smpl.: disease group biventricular HD simple. Table S2. Group definitions. Destinct groups are defined by both OPS and ICD codes, the respective codes are concatenated with a logical ‘OR’. OPS: German procedure coding system; ICD: internationial classification of diseases. Table S3. Number of patients per heart defect group stratified by the heart disease history and the deceased status. UVHD I: disease group univentricular heart defect (HD) 1; UVHD II: disease group univentricular HD 2; BVHD cmplx.: disease group biventricular HD complex; BVHD smpl.: disease group biventricular HD simple. N/A: not applicable, i.e. no previous hospitalization. Table S4. Dataset partitions into a training dataset (60%) and an independent holdout test dataset (40%). UVHD I: disease group univentricular heart defect (HD) 1; UVHD II: disease group univentricular HD 2; BVHD cmplx.: disease group biventricular HD complex; BVHD smpl.: disease group biventricular HD simple. Table S5. Feature importance. Mean absolute SHAP values for XGB and RSF. The feature importance was computed from the SHAP values that were averaged by feature and observation across all 100 repeated CV models for each ML method. The rank of each variable according to the computed SHAP values are given in brackets next to the SHAP value. Table S6. Results of the Cox Proportional Hazards model. Columns 2 and 3 present the median values and 95% conficence intervals (CI) for the hazard ratio (HR) and *p*-values of the 100 models from the repeated CV (weighted by the number of samples in the training folds). Columns 4 and 5 present the model output of the single Cox PH model fitted with the complete training dataset. The reference group of the variable ‘Disease group’ was univentricular heart defect (UVHD) I. HR: harzard ratio. CI: confidence interval. UVHD II: disease group univentricular heart defect (HD) 2; BVHD cmplx.: disease group biventricular HD complex; BVHD smpl.: disease group biventricular HD simple; Inf: infinite. Figure S1. Boxplot of the days until discharge after surgery stratified by disease group and deceased status. Dashed line: 99.5% percentile of the days until discharge computed over the whole sample. 0: censored; 1: deceased. UVHD I: disease group univentricular heart defect (HD) 1; UVHD II: disease group univentricular HD 2; BVHD cmplx.: disease group biventricular HD complex; BVHD smpl.: disease group biventricular HD simple. Figure S2. Performance: Boxplots to visualize the performance on the independent holdout test dataset per disease group for each algorithm. The underlying data for each boxplot is the performance of the 100 models from the repeated CV during validation and when applying these 100 models to predict the outcome in the holdout test dataset subsetted to the respective disease group. UVHD I: disease group univentricular heart defect (HD) 1; UVHD II: disease group univentricular HD 2; BVHD cmplx.: disease group biventricular HD complex; BVHD smpl.: disease group biventricular HD simple.
