# Supplementary material for: Using Machine Learning and Feature Importance to Identify Risk Factors for Mortality in Pediatric Heart Surgery
Source: Diagnostics (Basel). 2024 Nov 18;14(22):2587. doi: 10.3390/diagnostics14222587 (PMC11592497; doi:10.3390/diagnostics14222587)
Supplement: Supplementary file 1 [file diagnostics-14-02587-s001.zip › diagnostics-3255339-supplementary.pdf]

diagnostics

MDPI

Supplementary Materials

Using Machine Learning and Feature Importance to Identify Risk Factors for Mortality in Pediatric Heart Surgery

Lorenz A. Kapsner [1,2], Manuel Feißt [3], Ariawan Purbojo [4], Hans-Ulrich Prokosch [1], Thomas Ganslandt [1], Sven Dittrich [5], Jonathan M. Mang [6,*,†] and Wolfgang Wällisch [5,†]

[1] Medial Informatics, Friedrich-Alexander-Universität Erlangen-Nürnberg (FAU), 91058 Erlangen, Germany; lorenz.kapsner@uk-erlangen.de (L.A.K.); hans-ulrich.prokosch@uk-erlangen.de (H.-U.P.); thomas.ganslandt@fau.de (T.G.)
[2] Institute of Radiology, Universitätsklinikum Erlangen, Friedrich-Alexander-Universität Erlangen-Nürnberg (FAU), 91054 Erlangen, Germany
[3] Institute of Medical Biometry, University of Heidelberg, 69117 Heidelberg, Germany; feisst@imbi.uni-heidelberg.de
[4] Department of Paediatric Cardiac Surgery, Universitätsklinikum Erlangen, Friedrich-Alexander-Universität Erlangen-Nürnberg (FAU), 91054 Erlangen, Germany; ariawan.purbojo@uk-erlangen.de
[5] Department of Pediatric Cardiology, Universitätsklinikum Erlangen, Friedrich-Alexander-Universität Erlangen-Nürnberg (FAU), 91054 Erlangen, Germany; sven.dittrich@uk-erlangen.de (S.D.); wolfgang.waellisch@uk-erlangen.de (W.W.)
[6] Medical Center for Information and Communication Technology, Universitätsklinikum Erlangen, 91054 Erlangen, Germany
* Correspondence: jonathan.mang@uk-erlangen.de
† These authors contributed equally to this work.

Supplemental Methods

Data Imputation

The observed missing values in the dataset stratified by the training dataset and the test dataset are given in Table S2. A total of 3 deceased cases had a post-operative observed time of 0 days and most probably deceased during the surgery or shortly thereafter. For two of these cases, all values of the post-operative laboratory analytes were missing in the dataset. Therefore, these values were replaced with the corresponding pre-operative values instead of imputing them, in the sense of a "last-observation-carried-forward" (LCOF) imputation. The remaining missing values were imputed with multivariate imputation by chained equations using the 'mice' R package[1], which was repeated 10 times. To receive one complete dataset for carrying out the experiments, the imputed datasets were merged. The imputation model was trained with the observations of the training dataset only in order to avoid a potential leaking of information from the training data into the independent holdout test dataset.

Machine Learning Experiments

In order to ensure that the hyperparameters of the different ML methods were optimized on the same identical CV folds, a vector containing interactions between the survival outcome and the disease group variable was computed before carrying out the experiments, using the 'multi_strata()' function from the 'splitTools' R package[2]. Setting the same seed for all experiments of the different ML methods ensured a deterministic stratified fold generation throughout the experiments.

Supplemental Results

Comparison with CPH

For comparison with a standard statistical approach, the CPH regression models were fitted in the same experimental setup as the ML methods using only a subset of the available features, namely the union set of the 5 most important features as previously identified by XGB and RSF. Columns 2 and 3 of Table S6 show the median hazard ratios and p-values of the 100 CPH models from the repeated CV. For comparison, columns 4 and 5 show the results of a single CPH regression model that was fitted using the complete training dataset.

According to the results of the CPH models, only the *disease group* was statistically significant associated with the mortality risk after surgery for CHD in pediatric patients. The repeated CV results (columns 2 and 3 of Table S6) show a significantly decreased hazard ratio (HR) for the disease groups *UVHD II* (median HR [95%-CI]: 0.119 [0.044; 0.200]), *BVHD cmplx.* (median HR [95%-CI]: 0.423 [0.331; 0.552]), and *BVHD smpl.* (median HR [95%-CI]: 0.083 [0.047; 0.124]) in comparison with cases from the disease group *UVHD I*. The validity of this experimental approach is underlined on the one hand by the observation that the results of the CPH 10×10 repeated CV models align well with those from the single CPH model fitted with the complete training dataset (columns 4 and 5 of Table S6), and on the other hand, the CPH results align with the ML results.

Supplemental Tables

Table S1. Inclusion criteria. If groups are defined by both OPS and ICD codes, the respective codes are concatenated with a logical 'AND' between code systems (ICD/OPS) and 'OR' within one code system. OPS: German procedure coding system; ICD: internationial classification of diseases; UVHD I: disease group univentricular heart defect (HD) 1; UVHD II: disease group univentricular HD 2; BVHD cmplx.: disease group biventricular HD complex; BVHD smpl.: disease group biventricular HD simple.

Disease group	Heart failure (subcategory)	OPS codes	ICD codes	Anamnesis
UVHD Ia	Norwood-Typ-I (HLHS)	5-359.66, 5-359.67	Q23.4	
	Norwood-Typ-I (with aortic arch stenosis)	5-359.66, 5-359.67	Q25.1, Q25.2, Q25.4	
	Norwood-Typ-I (without aortic arch stenosis)	5-359.66, 5-359.67		
UVHD Ib	Univentricular heart	5-390	Q20.4, Q22.4, Q23.4, Q25.2	None of these OPS: 5-359.60, 5-359.61, 5-359.62, 5-359.63, 5-359.64, 5-359.65, 5-359.66, 5-359.67
UVHD IIa	Glenn + Norwood in anamnesis	5-359.60, 5-359.61, 5-359.62		OPS: 5-359.66, 5-359.67
	Glenn + Palliation with Shunt/Banding in anamnesis	5-359.60, 5-359.61, 5-359.62		OPS: 5-390
	Glenn without surgery	5-359.60, 5-359.61, 5-359.62		
	Fontan + Norwood in anamnesis	5-359.63, 5-359.64, 5-359.65		OPS: 5-359.66, 5-359.67
	Fontan + Shunt in anamnesis	5-359.63, 5-359.64, 5-359.65		OPS: 5-390
	Other correction	5-359.6x		
	Fontan (Glenn)	5-359.63, 5-359.64, 5-359.65		OPS: 5-359.60, 5-359.61, 5-359.62

	Fontan without recorded surgery	5-359.63, 5-359.64, 5-359.65		
UVHD IIb	Glenn and/or Fontan with other surgery	5-35, 5-36, 5-37, 5-38, 5-39		OPS: 5-359.60, 5-359.61, 5-359.62, 5-359.63, 5-359.64, 5-359.65
BVHD cmplx.	Complex biventricular	5-356.6		
	Complex biventricular	(5-359.0) AND (5-356.7)	Q21.2	
	Complex biventricular	(5-359.0) AND (5-356.8)	Q21.2	
	Complex biventricular	5-351.0c, 5-351.0e, 5-357.7, 5-357.8, 5-358.07, 5-358.0e, 5-359.1, 5-359.20, 5-359.4, 5-359.5, 5-359.8, 5-359.x, 5-390		
	Complex biventricular	5-350, 5-351, 5-352, 5-353, 5-354, 5-355, 5-356, 5-357.0, 5-357.1, 5-357.2, 5-357.3, 5-357.4, 5-357.5, 5-357.6, 5-357.9, 5-357.x, 5-357.y, 5-358, 5-359.0, 5-359.21, 5-359.3, 5-359.7	Q20.0, Q20.3, Q20.5, Q20.6, Q20.8, Q22.0, Q22.5, Q22.6, Q23.8, Q25.2, Q25.5	
BVHD smpl.	Uncomplicated biventricular	5-350, 5-351, 5-352, 5-353, 5-354, 5-355, 5-356, 5-357.0, 5-357.1, 5-357.2, 5-357.3, 5-357.4, 5-357.5, 5-357.6, 5-357.9, 5-357.x, 5-357.y, 5-358, 5-359.0, 5-359.21, 5-359.3, 5-359.7		

Table S2. Group definitions. Destinct groups are defined by both OPS and ICD codes, the respective codes are concatenated with a logical 'OR'. OPS: German procedure coding system; ICD: internationial classification of diseases.

Group	OPS codes	ICD codes
Malformations	-	D80-D89, Q00-Q07, Q30-Q39, Q40-Q45, Q50-Q52, Q60-Q64, Q77-Q79, Q80-Q89
Chrom. alterations	-	Q90-Q99
Pulm. hypertension	-	I27.8
Open thorax	5-916.7a	M96.80

Table S3. Number of patients per heart defect group stratified by the heart disease history and the deceased status. UVHD I: disease group univentricular heart defect (HD) 1; UVHD II: disease group univentricular HD 2; BVHD cmplx.: disease group biventricular HD complex; BVHD smpl.: disease group biventricular HD simple. N/A: not applicable, i.e. no previous hospitalization.

Status	Disease group	Heart disease history	N
censored	UVHD I	UVHD Ib	3
		BVHD cmplx.	3
		N/A	13
	UVHD II	UVHD Ia	55
		UVHD Ib	26
		BVHD cmplx.	10
		UVHD IIa / UVHD IIb	5
		N/A	8
	BVHD cmplx.	UVHD Ia	4
		UVHD Ib	4
		BVHD cmplx.	56
		BVHD smpl.	9
		N/A	190
	BVHD smpl.	UVHD Ib	2
		BVHD cmplx.	54
		BVHD smpl.	43
		N/A	745
deceased	UVHD I	UVHD Ib	2
		N/A	29
	UVHD II	UVHD Ia	3
		UVHD Ib	2
		BVHD cmplx.	1
		N/A	1
	BVHD cmplx.	BVHD cmplx.	7
		N/A	21
	BVHD smpl.	BVHD cmplx.	1
		N/A	5

Table S4. Dataset partitions into a training dataset (60%) and an independent holdout test dataset (40%). UVHD I: disease group univentricular heart defect (HD) 1; UVHD II: disease group univentricular HD 2; BVHD cmplx.: disease group biventricular HD complex; BVHD smpl.: disease group biventricular HD simple.

	**Training Dataset**	**Training Dataset**	**Training Dataset**	**Holdout Test Dataset**	**Holdout Test Dataset**	**Holdout Test Dataset**
Diagnosis group	Cencored	Deceased	Total	Cencored	Deceased	Total
UVHD I	13 (41.9 %)	18 (58.1 %)	31 (100 %)	6 (31.6 %)	13 (68.4 %)	19 (100 %)
UVHD II	62 (95.4 %)	3 (4.6 %)	65 (100 %)	42 (91.3 %)	4 (8.7 %)	46 (100 %)
BVHD cmplx.	155 (90.1 %)	17 (9.9 %)	172 (100 %)	108 (90.8 %)	11 (9.2 %)	119 (100 %)
BVHD smpl.	508 (99.2 %)	4 (0.8 %)	512 (100 %)	336 (99.4 %)	2 (0.6 %)	338 (100 %)
Total	738 (94.6 %)	42 (5.4 %)	780 (100 %)	492 (94.3 %)	30 (5.7 %)	522 (100 %)

Table S5. Feature importance. Mean absolute SHAP values for XGB and RSF. The feature importance was computed from the SHAP values that were averaged by feature and observation across all 100 repeated CV models for each ML method. The rank of each variable according to the computed SHAP values are given in brackets next to the SHAP value.

Variable	XGB	RSF
Serum creatinine (maximum)	0.383 (1)	0.313 (1)
Disease group	0.203 (2)	0.188 (3)
Urea (maximum)	0.153 (3)	0.132 (7)
Open thorax	0.127 (4)	0.133 (6)
Age at surgery	0.116 (5)	0.179 (5)
Circulatory arrest during surgery	0.101 (6)	0.109 (9)
Aortic cross clamp time	0.09 (7)	0.186 (4)
Days between admission and surgery	0.086 (8)	0.236 (2)
C-reactive protein (maximum)	0.069 (9)	0.123 (8)
Leukocytes (minimum)	0.05 (10)	0.072 (12)
Malformations	0.033 (11)	0.031 (14)
Sex	0.031 (12)	0.077 (11)
Heart lung machine during surgery	0.01 (13)	0.021 (17)
Hypothermia during surgery	0.006 (14)	0.025 (16)
No. of previous admissions	0.005 (15)	0.089 (10)
Heart disease history	0.004 (16)	0.029 (15)
Chrom. alterations	0.003 (17)	0.005 (18)
Weight < 2500 g	0.002 (18)	0.046 (13)
Pulm. hypertension	0 (19)	0.001 (19)

Table S6. Results of the Cox Proportional Hazards model. Columns 2 and 3 present the median values and 95% conficence intervals (CI) for the hazard ratio (HR) and p-values of the 100 models from the repeated CV (weighted by the number of samples in the training folds). Columns 4 and 5 present the model output of the single Cox PH model fitted with the complete training dataset. The reference group of the variable 'Disease group' was univentricular heart defect (UVHD) I. HR: harzard ratio. CI: confidence interval. UVHD II: disease group univentricular heart defect (HD) 2; BVHD cmplx.: disease group biventricular HD complex; BVHD smpl.: disease group biventricular HD simple; Inf: infinite.

**Predictors**	**Median HR (95%-CI)**	**Median p-value (95%-CI)**	**HR (95%-CI)**	**p-value (95%-CI)**
Age at surgery	1.000 [0.999; 1.000]	0.837 [0.398; 0.995]	1.000 [0.999; 1.001]	0.816
Aortic cross clamp time	0.995 [0.993; 0.997]	0.124 [0.026; 0.360]	0.995 [0.990; 1.001]	0.104
Serum creatinine (maximum)	1.760 [1.292; 2.806]	0.148 [0.019; 0.522]	1.765 [0.848; 3.673]	0.129
Days between admission and surgery	0.977 [0.950; 0.985]	0.368 [0.209; 0.496]	0.976 [0.928; 1.027]	0.344
Disease group [UVHD II]	0.119 [0.044; 0.200]	0.015 [0.003; 0.075]	0.117 [0.022; 0.610]	0.011
Disease group [BVHD cmplx.]	0.423 [0.331; 0.552]	0.040 [0.009; 0.159]	0.426 [0.195; 0.929]	0.032
Disease group [BVHD smpl.]	0.083 [0.047; 0.124]	0.000 [0.000; 0.002]	0.079 [0.022; 0.280]	<0.001
Open thorax [yes]	1.337 [0.910; 1.834]	0.510 [0.184; 0.986]	1.296 [0.569; 2.953]	0.537
Urea (maximum)	1.008 [1.001; 1.018]	0.383 [0.056; 0.899]	1.008 [0.991; 1.026]	0.332
Observations			780	
R^2 Nagelkerke			0.164	

Supplemental Figures

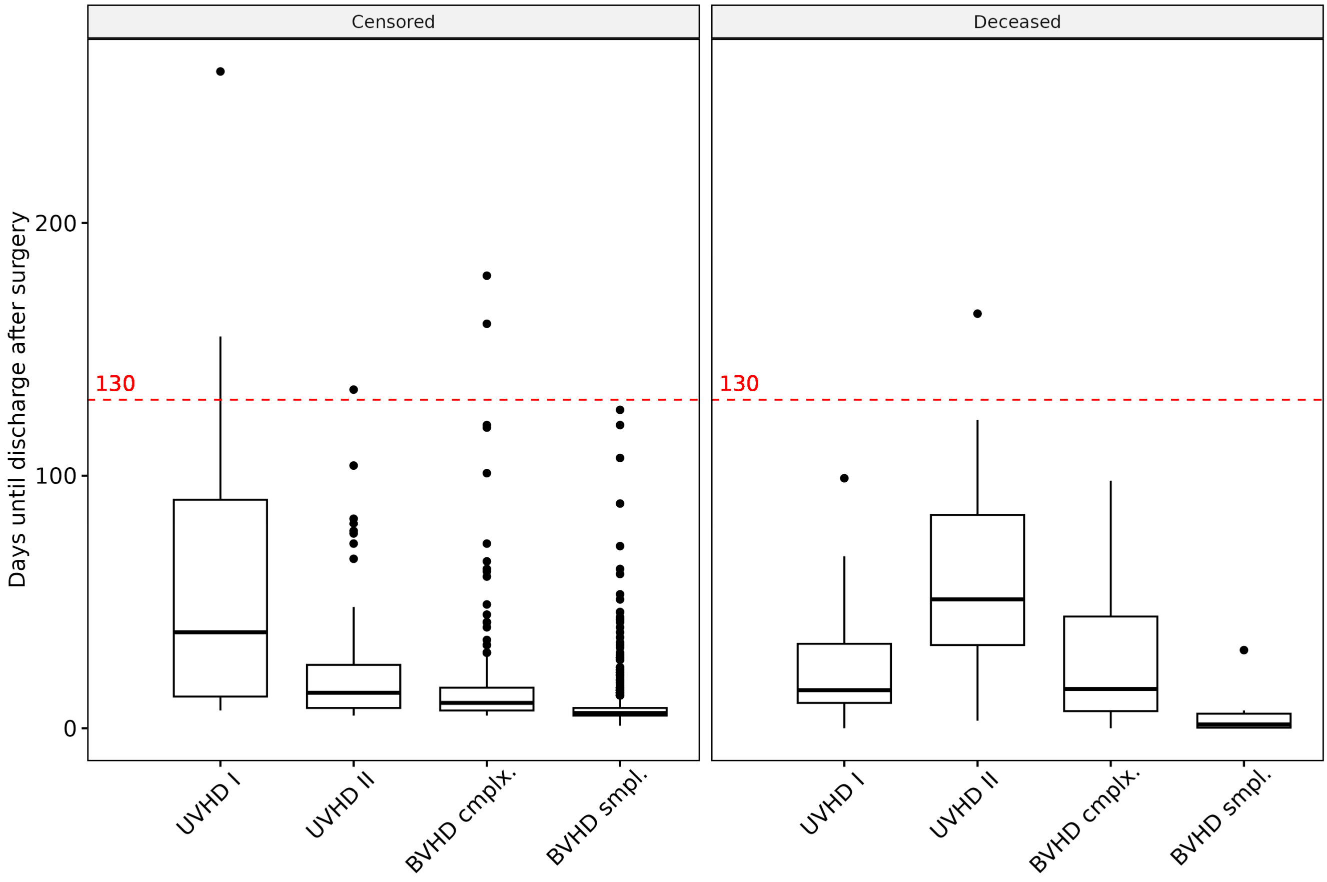

Figure S1. Boxplot of the days until discharge after surgery stratified by disease group and deceased status. Dashed line: 99.5% percentile of the days until discharge computed over the whole sample. 0: censored; 1: deceased. UVHD I: disease group univentricular heart defect (HD) 1; UVHD II: disease group univentricular HD 2; BVHD cmplx.: disease group biventricular HD complex; BVHD smpl.: disease group biventricular HD simple.

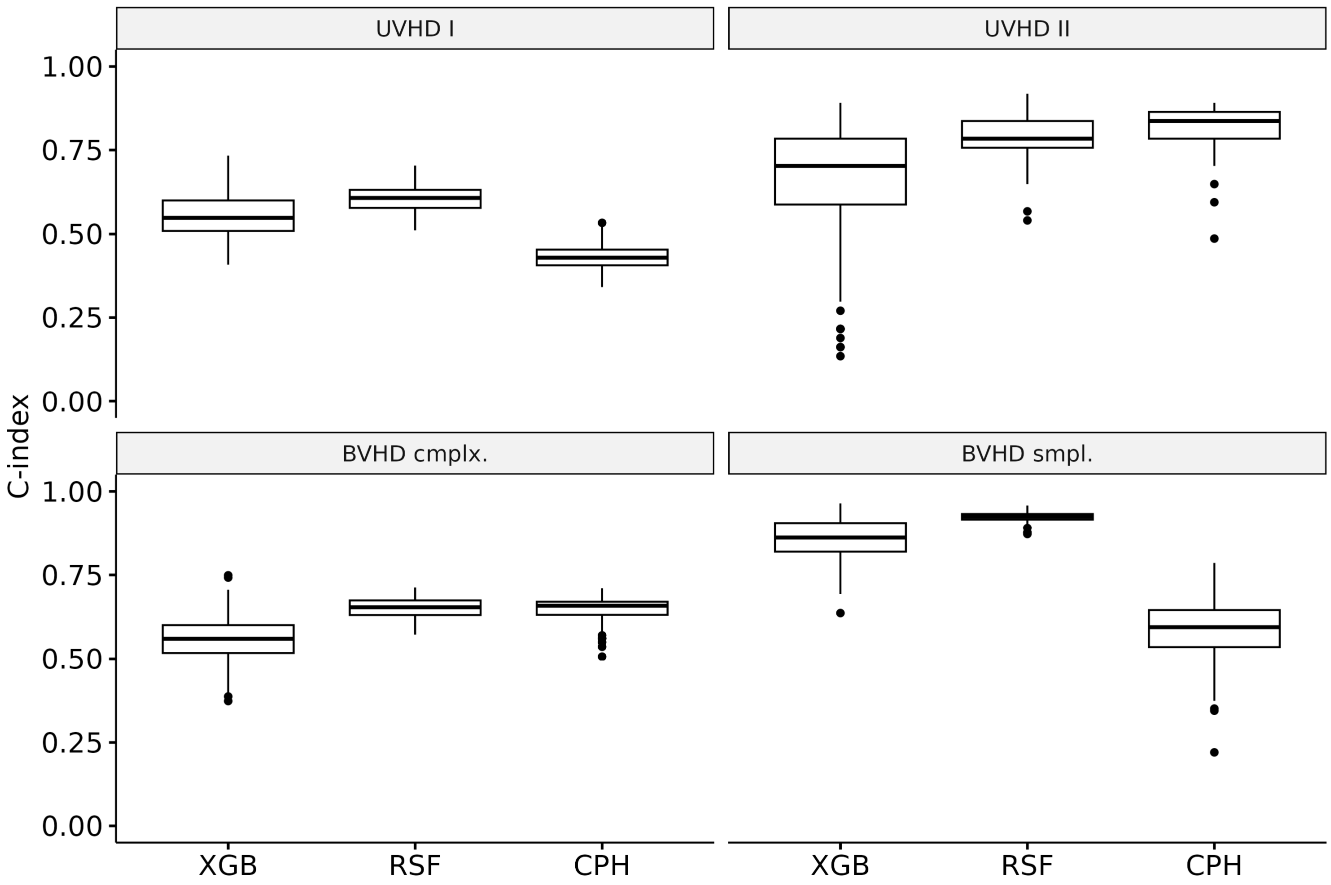

Figure S2. Performance: Boxplots to visualize the performance on the independent holdout test dataset per disease group for each algorithm. The underlying data for each boxplot is the performance of the 100 models from the repeated CV during validation and when applying these 100 models to predict the outcome in the holdout test dataset subsetted to the respective disease group. UVHD I: disease group univentricular heart defect (HD) 1; UVHD II: disease group univentricular HD 2; BVHD cmplx.: disease group biventricular HD complex; BVHD smpl.: disease group biventricular HD simple.

References

1. Buuren, S. van & Groothuis-Oudshoorn, K. Mice: Multivariate imputation by chained equations in r. **45**, 1–67 (2011).
2. Mayer, M. splitTools: Tools for data splitting. (2022).
